# Pre- and intratherapeutic predictors of overall survival in patients with advanced metastasized castration-resistant prostate cancer receiving Lu-177-PSMA-617 radioligand therapy

**DOI:** 10.1186/s12894-022-01050-3

**Published:** 2022-07-04

**Authors:** Robin Wrenger, Michael Jüptner, Marlies Marx, Yi Zhao, Maaz Zuhayra, Amke Caliebe, Daniar Osmonov, Ulf Lützen

**Affiliations:** 1 Department of Nuclear Medicine, Molecular Diagnostic Imaging and Therapy, University Hospital of Schleswig-Holstein (UKSH), Campus Kiel, Arnold-Heller-Straße 3, Haus L, 24105 Kiel, Germany; 2 Institute of Medical Informatics and Statistics, Kiel University and University Hospital of Schleswig-Holstein (UKSH), Kiel, Germany; 3 Department of Urology and Pediatric Urology, University Hospital of Schleswig-Holstein (UKSH), Kiel, Germany

**Keywords:** Lu-177-PSMA-617, Radioligand therapy, castration-resistant prostate cancer, Survival analysis

## Abstract

**Background:**

Systemic Lutetium-177 prostate-specific membrane antigen-617 radioligand therapy (Lu-177-PSMA-617-RLT) is a novel treatment approach in patients suffering from metastasized castration-resistant prostate cancer. Nonetheless, a therapeutic response may fail to appear in a proportion of patients. This study aims to identify routinely obtainable pre- and intratherapeutic parameters to allow a prediction of overall survival in patients receiving Lu-177-PSMA-617 radioligand therapy.

**Methods:**

Between January 2015 and December 2020 52 patients treated with a total of 146 cycles Lu-177-PSMA-617-RLT were retrospectively analysed in a single-center trial. The median overall survival time (OS) was compared to pre-therapeutic serological parameters, the extend of metastatic spread and previously performed therapies using Kaplan–Meier estimators and multivariate Cox-regression. Bonferroni-Holm correction was performed on all statistical tests.

**Results:**

The median OS of all patients was 55.6 weeks. Multivariate Cox-regression revealed significant lower survival for decreased pretherapeutic hemoglobin levels (HR 0.698 per g/dl; 95%-CI 0.560–0.872; *p* = 0.001), increased lactate dehydrogenase (LDH) levels (HR 1.073 per 25 U/l; 95%-CI 1.024–1.125; *p* = 0.003) and the presence of hepatic metastasis (HR 6.981; 95%-CI 2.583–18.863; *p* < 0.001). Increased pretherapeutic c-reactive protein (CRP), alkaline phosphatase (ALP) and gamma-glutamyltransferase (GGT) levels were also associated with a shorter survival. A prostate-specific antigen decline after one therapy cycle did not significantly correlate with an increased survival. No significant relations were observed between overall survival time and other serological parameters or previously performed therapies.

**Conclusion:**

Pre-therapeutic hemoglobin and LDH levels, as well as the presence of hepatic metastasis are independent predictors of overall survival in patients receiving Lu-177-PSMA-617-RLT. CRP, ALP and GGT levels cloud be utilized as additional decision aids when a Lu-177-PSMA-617-RLT is intended.

*Trial Registration *Not applicable (retrospective observational study).

**Supplementary Information:**

The online version contains supplementary material available at 10.1186/s12894-022-01050-3.

## Background

Since the introduction of systemic Lu-177-PSMA-617 radioligand therapy (RLT) and the first description of a successful treatment in 2015 [1], this novel treatment approach continues to gain importance in patients suffering from metastasized castration-resistant prostate cancer (mCRPC). Multiple clinical trials demonstrated a low profile of side effects, favourable safety results and high rates of treatment response [2–6]. Therefore, this therapeutic procedure is currently discussed as a “cornerstone” or a “game changer” in the therapeutic sequence of patients with metastasized prostate cancer [7, 8].

The recently conducted multicentre, open-label, phase-III VISION trial [9] demonstrated a prolonged imaging-based progression-free survival and a longer overall survival in a large cohort of patients, receiving Lu-177-PSMA-617 radioligand therapy. These results may indicate a major therapeutic potential in the future treatment of patients with advanced prostate cancer.

Nonetheless, a therapeutic response may fail to appear in a proportion of patients. A meta-analysis revealed a pooled rate of non-response to this treatment procedure in 32% of the patients [10], i.e., no PSA decline was observed after the first treatment cycle. Therefore, it is of major importance to identify reliable prognostic factors for the prediction of treatment response prior to the initiation of the therapy. The prognostic implications of a pretherapeutic Ga-68-PSMA PET/CT, as well as its role in response assessment within a Lu-177-PSMA-617 therapy regime, have been well described by several clinical investigations [11–15].

Evaluating predictive parameters for sufficient treatment monitoring, a post-therapeutic PSA decline of ≥ 50%, as well as a PSA decline of any height after the first treatment cycle compared to baseline levels during follow-up has been identified and consistently been confirmed as the most important marker indicating treatment response [16, 17].

However, since the course of PSA as a marker of response is only useful in the post-therapeutic monitoring after the initiation of the first treatment cycle, there are no consistent pre- and intratherapeutic factors for the prediction of overall survival yet.

## Methods

### Study aim, design, and participants

This study aims to identify routinely obtainable, and easily accessible pre- and intratherapeutic predictive factors for overall survival in a monocentric patient cohort treated with Lu-177-PSMA-617-RLT for metastasized castration resistant prostate cancer.

Overall, 56 patients suffering from mCRPC and periodically treated with Lu-177-PSMA-617-RLT at the University Hospital of Kiel, Germany, between January 2015 and December 2020 were enrolled in this observational retrospective study.

Interruption of the therapy regimen, or application of one or more therapy cycles elsewhere than in the University Hospital of Kiel, led to exclusion from the study.

By the end of the observation period, 4 individuals were excluded from the study. The remaining 52 patients received a total of 146 cycles of Lu-177-PSMA-617-RLT.

Lu-177-PSMA-617-RLT was applied as individual compassionate use according to the common regimen described in the national consensus advice [18].

The study was performed in accordance with the ethical standards laid down in the 1964 Declaration of Helsinki and its subsequent revisions and has been approved by the institutional review board (AZD410/21). Written informed consent was obtained from all subjects.

### Data collection

We collected baseline patient characteristics, including previously performed systemic and local-regional treatments, the extent of metastatic spread and the number of applied Lu-177-PSMA-617-RLT cycles. A detailed list of the patient characteristics is shown in Table 1.

**Table 1:** Detailed patient characteristics of 52 patients treated with Lu-177-PSMA-617 RLT

Characteristic	Subgroup	No. of patients	Percentage
Number of treatment cycles applied	1 treatment cycle	15/52	28.8
	2 treatment cycles	11/52	21.2
	3 treatment cycles	5/52	9.6
	4 treatment cycles	16/52	30.8
	5 treatment cycles	1/52	1.9
	6 treatment cycles	3/52	5.8
	7 treatment cycles	1/52	1.9
Systemic pretreatment prior to Lu-177-PSMA-617 radioligand therapy	Bicalutamide and/or Leuprorelin	48/52	92.3
	Enzalutamide and/or Abiraterone	44/52	84.6
	Chemotherapy (Docetaxel or Cabazitaxel)	39/52	75.0
	Ra-223-Dichloride	8/52	15.4
Local-regional pretreatment prior to Lu-177-PSMA-617 radioligand therapy	Radical prostatectomy	33/52	63.5
	External beam radiation and/or Brachytherapy	39/52	75.0
Location of metastases in pre-therapeutic Ga-68-PSMA-PET/CT scan	Skeletal	52/52	100.0
	Lymphatic	42/52	80.8
	Hepatic	9/52	17.3
	Cerebral	3/52	5.8
	Pulmonary	3/52	5.8

	Median	Range
Age at 1st treatment cycle	72.1 years	56.3–84.6 years

Blood parameters were measured one day prior to the first injection of Lu-177-PSMA-617, thus defined as baseline level and one day prior to any following Lu-177-PSMA-617 therapy cycle. Measured laboratory data included full blood count, electrolytes, renal and liver function panel. All obtained blood parameters and related abbreviations are listed in Additional file 1: Table S1. The overall survival time (OS) was defined as the time span from the day of the first injection of Lu-177-PSMA-617 up to death of any cause.

### Data analysis

In our primary analysis, we assessed the impact of systemic and local-regional pre-treatment and the location of metastases prior to the first therapy cycle on the overall survival time. Additionally, elevated baseline lactate dehydrogenase (LDH), alkaline phosphatase (ALP), gamma-glutamyl transferase (GGT) and c-reactive protein (CRP) levels above their reference range with an upper cut-off value of 250 U/l, 130 U/l, 60 U/l and 5 mg/l were used for comparison of the overall survival time. Patients with preexisting anemia were compared to those having normal baseline hemoglobin levels. We defined anaemia at a cut-off value of hemoglobin (HB) < 10 g/dl in accordance with another study investigating important factors for survival in patients with hormone-refractory prostate cancer treated with chemotherapy [19].

In a secondary analysis, intratherapeutic data were evaluated.

We examined the change in serum PSA level to determine the therapeutic response. A decrease of ≥ 50% from baseline, measured at least 3 weeks after the first treatment cycle, was considered as a positive therapeutic response. We compared the overall survival in patients showing a positive therapeutic PSA response versus those who did not meet the criteria for therapeutic response. Moreover, we compared patients without any PSA decline below their baseline versus patients responding with equal/increasing PSA levels. Survival time of patients that received only one or two therapy cycles was compared to those that received more than two cycles.

Finally, we evaluated the change of laboratory parameters within the therapy regimen in sense of an explorative analysis.

Kaplan–Meier plots were created for graphical presentation of the overall survival time. Differences between two groups were tested using the log-rank test. For those variables that revealed a statistically significant difference in survival time during the primary analysis, an additional multivariate Cox-regression with backwards selection was performed.

The significance level was set at 0.05. To counteract the multiple testing problem, Bonferroni-Holm correction was used on all 22 performed statistical tests. An asterisk (*) following an *p*-value indicates that the *p*-value remained significant after adjusting for multiple testing. All statistical calculations and figures were performed/created using SPSS 27 statistics software (IBM, NY, USA). Figures were edited using Adobe Illustrator CC 2021 (Adobe, CA, USA).

## Results

Out of the 52 patients included in this analysis, 32 patients (61.5%) died during the observation period, with the earliest event after 9.7 weeks and the latest death after 159.9 weeks. In all patients who died during the observation period, death was related to the metastasized prostate malignancy. The observation period of the patients still alive ranged from 4 to 179 weeks.

**Fig. 1:**
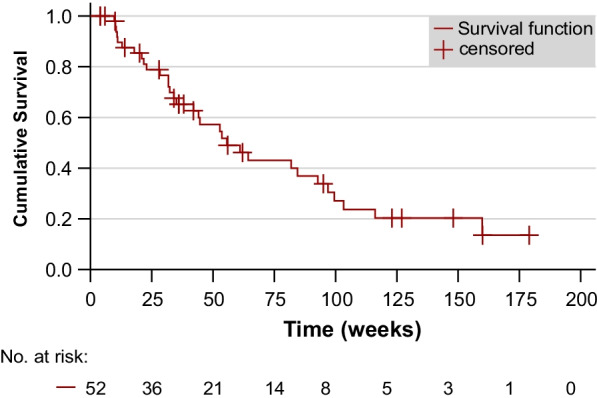
Overall survival time of the patients (n = 52)

The median survival time calculated for all patients was 55.6 weeks (95%-CI 34.2–77.0 weeks, see Fig. 1).

A mean of 2.8 therapy cycles were applied per patient. The median amount of activity administered per cycle was 6.0 GBq, ranging from 5.0 to 7.0 GBq.

None of the previously applied treatments in our patients’ history revealed a significant effect on the overall survival. Prior to the adjustment for multiple testing the use of the androgen receptor inhibitors Enzalutamide/Arbiraterone revealed a significant increase in overall survival (median 116.1 vs. 52.7 weeks, *p* = 0.018), whereas the distribution of the patients between those groups was inhomogenous. Additionally, the application of a cytotoxic chemotherapy prior to the Lu-177-PSMA-617-RLT was associated with a shorter overall survival time compared to patients who were unfit for chemotherapy or refused this type of treatment (53.6 vs. 103.3 weeks). However, this association did miss significance (*p* = 0.091).

**Fig. 2:**
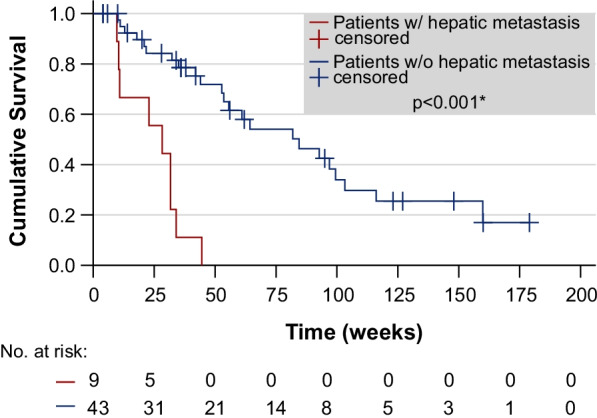
Overall survival time compared in patients with documented hepatic metastases versus patients without hepatic metastasis

Overall survival was strongly reduced in patients suffering from hepatic metastases (median survival time 28.3 weeks vs. 84.4 weeks, *p* < 0.001*, see Fig. 2), whereas other sites of metastatic spread showed no significant difference. At initiation of the therapy regimen, 43 patients presented with multiple sites of metastatic disease, whereas 9 patients presented with bone metastasis only. A significant difference in survival could not be shown when comparing these two groups (61.0 weeks vs. 55.6 weeks, *p* = 0.712). Detailed results of the patients’ premedication and metastasis are presented in Additional file 2: Table S2.

**Fig. 3:**
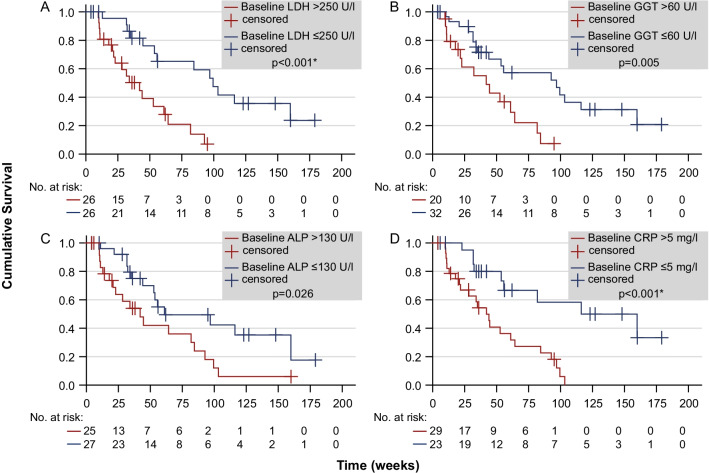
Overall survival time regarding elevated baseline LDH (**A**), GGT (**B**), ALP (**C**) and CRP (**D**) levels at threshold > 250 U/l, > 60 U/l, > 130 U/l and > 5 mg/l, respectively

The impact of increased baseline laboratory parameters on overall survival is presented in Table 2, the corresponding Kaplan–Meier graphs are shown in Fig. 3.

**Table 2:** Kaplan–Meier estimator depending on baseline hemoglobin, LDH, GGT, ALP and CRP level within/outside the set reference range

Baseline parameter	Reference range	Median survival within reference range	Median survival outside reference range	*p* value
Hemoglobin	≥ 10 g/dl	81.9 (n = 40)	44.0 (n = 12)	0.035
LDH	≤ 250 U/l	99.4 (n = 26)	42.0 (n = 26)	< 0.001*
GGT	≤ 60 U/l	96.9 (n = 32)	42.0 (n = 20)	0.005
ALP	≤ 130 U/l	61.0 (n = 27)	42.0 (n = 25)	0.026
CRP	≤ 5 mg/l	116.1 (n = 23)	42.0 (n = 29)	< 0.001*

*LDH*, lactate dehydrogenase; *GGT*, gamma-glutamyl transferase; *ALP*, alkaline phosphatase; *CRP*, c-reactive protein; (*) statistically significant after adjusting for multiple testing

There was a strong discrepancy in survival time of the patients presenting with increased baseline LDH level > 250 U/l (42.0 weeks vs. 99.4 weeks, *p* < 0.001*) and elevated CRP levels > 5 mg/l (42.0 vs. 116.1 weeks, *p* < 0.001*). Likewise, elevated ALP and GGT levels were associated with a decrease of median survival time, although these results were less distinct and did not reach statistical significance.

After adjustment for multiple testing the comparison between anaemic and non-anaemic patients showed no significant difference in median overall survival time at the cut-off value of 10 g/dl (44.0 vs. 81.9 weeks, *p* = 0.035, see Additional file 3: Figure S1).

Hemoglobin, LDH, ALP, GGT, CRP and the presence of hepatic metastasis were included in the multivariate Cox-regression, as these variables showed statistically significant results in the uncorrected univariate analysis. Multivariate Cox-regression revealed hemoglobin, LDH levels and the presence of liver metastases as significant covariates (see Table 3).

**Table 3:** Multivariate cox regression for correlation analysis between baseline parameters and overall survival

	Multivariate cox-regression		
Covariates	HR	95% CI	*p* value
Hepatic metastasis (if present)	6.981	2.583–18.863	< 0.001*
Hemoglobin (per g/dl)	0.698	0.560–0.872	0.001*
LDH (per 25 U/l)	1.073	1.024–1.125	0.003*
GGT (per U/l)	†	†	†
ALP (per U/l)	†	†	†
CRP (per mg/l)	†	†	†

*HR*, Hazard ratio; 95% *CI*, 95% confidence interval

^†^corresponding covariate was removed during backwards selection; (*) statistically significant after adjusting for multiple testing

Hemoglobin levels were associated with overall survival in sense of a negative correlation (HR 0.698; *p* = 0.001*). A hazard ratio of 1.073 was calculated for each 25 U/l increase in baseline LDH (*p* = 0.003*). The presence of hepatic metastases proved to be the poorest prognostic factor with a hazard ratio (HR) of 6.981 if present (*p* < 0.001*).

A positive therapeutic PSA response was observed in 16 patients accompanied with a median survival of 96.9 weeks. The comparison with the non-responder group revealed no significant difference in overall survival (81.9 vs. 96.9 weeks, *p* = 0.072, see Additional file 4: Figure S2A). In contrast, 26 patients presented with a PSA decline of any quantity, which revealed an increase in survival (55.6 vs. 99.4 weeks, *p* = 0.015, see Additional file 4: Figure S2B). However, after Bonferroni-Holm correction this result did not remain statistically significant.

Comparison of the patients who received only one or two cycles of Lu-177-PSMA-617-RLT with those who received more than two therapy cycles showed a significant difference in overall survival with median 28.3 vs. 99.4 weeks (*p* < 0.001*, see Additional file 5: Figure S3).

We observed a continuous decrease in leucocyte, erythrocyte and platelet counts during the treatment period which persisted until the third treatment cycle. Leucocytes and platelets dropped about 22% from their baseline counts, whereas the decrease in erythrocyte count was less severe (4.68%). Coagulation parameters as well as electrolytes did not change notably during all treatment cycles. Creatinine, Urea, Glucose, Uric acid, alanine aminotransferase, GGT and total Protein level fluctuated without a distinct pattern. Regarding LDH, CRP and PSA levels, we noticed a drop of about 7%-50% from baseline levels in early cycles followed by a rebound during later cycles, partially even exceeding their baseline levels. The only parameter measured with a consistent decrease during all treatment cycles were ALP levels.

## Discussion

To date, Lu-177-PSMA-617-RLT is still applied with a palliative intention. Our result of 55.6 weeks median overall survival time is comparable to similar findings described in the literature [4, 9, 20].

Our findings suggest a major relevance of pre-therapeutic blood parameters and the presence of hepatic metastases in the prognosis of overall survival. Increased pre-therapeutic LDH levels were strongly associated with reduced overall survival time, indicating a poor prognosis. This result is consistent with previously published studies by Rathke et al. [21] and Heck et al. [22].

Liu et al. [23] and Li et al. [24] confirmed the relation between elevated CRP/ALP levels and poorer survival, Takemura et al. [25] concluded elevated GGT to be associated with shorter OS in men with mCRPC receiving Enzalutamide. In our study, however, CRP, ALP and GGT showed no additional effect to LDH, hemoglobin and hepatic metastasis in the multivariate Cox regression.

In the daily routine, in particular the upper cut-off value of the LDH reference range (250 U/l) could serve as a useful tool for overall survival prognosis in the clinical assessment of the patients prior to the initiation of the treatment. CRP, ALP and GGT may be used as additional indicators to determine the individual burden of disease, though, a potential confounding should be recognised.

Multivariate Cox-Regression suggests a negative correlation of increasing hemoglobin levels with overall survival time. However, we did not distinguish patients regarding the application of blood transfusions prior to the treatment.

The site and extend of metastatic spread should be taken into account when a Lu-177-PSMA-617-RLT is intended. Especially the presence of liver metastases was identified as the strongest factor associated with survival time reduction and has therefore a substantial impact on the prognosis. Since all our patients presented with manifest bone metastases, typically irregular and complex to demarcate from surrounding lesions, we were not able not perform a reliable further analysis of overall survival within this subgroup.

After correction for multiple testing, we did not detect a significant relationship between previously performed therapies and overall survival. Although the use of Enzalutamide/Abiraterone indicates a prolonged survival, it must be emphasized that this finding might be a random result due to the limitation in group size, as described earlier in this paper. Hence, we didn’t include this variable in the Cox-regression. Former application of cytotoxic chemotherapy prior to Lu-177-PSMA-617-RLT indicated a poorer survival among our patients. Likewise, Ahmadzadehfar et al. [26] also reported a longer overall survival in chemotherapy naïve patients in a significantly larger patient cohort. Thus, we share the conclusion of Barber et al. [27] suggesting that further clinical trials are necessary to identify the optimal time point for initiation of the Lu-177-PSMA-RLT in the treatment algorithm of advanced prostate cancer.

The proportion of patients with a PSA decline of any height from their baseline levels increased until the third therapy cycle, reaching 70.3% after one cycle, 76.9% after two cycles and 90.5% after three cycles. This indicates a delayed therapeutic response in a noteworthy subgroup of the patients. Comparable findings were described earlier by Rahbar et al. [2]. In consequence, we concluded that the absence of decreasing PSA levels after two treatment cycles should not result in preliminary withdrawal of the treatment, as biochemical response might first be measured after multiple treatment cycles.

A PSA drop of ≥ 50% after the first therapy cycle did not reveal a significant benefit in terms of survival. A drop of any height in PSA levels after the first treatment cycle is accompanied by an increased survival time span, however, this finding failed to reach significance after adjusting for multiple testing. In conclusion, a significant prediction on the survival of our patients regarding the change of PSA level after the first therapy cycle could not be verified.

In 12 patients (23.1%) death occurred after receiving only a single treatment cycle, either in consequence of blood count changes with increasing thrombocytopenia or because of rapidly decreasing health condition. These observations emphasize the limitations of Lu-177-PSMA-617-RLT in patients with decompensated end-stage disease. In those cases, systemic radioligand therapy seems to be ineffective in prolonging the patients’ life span and might even place additional stress on the patients at the end of their lives.

It appears trivial that the number of applied treatment cycles correlates strongly with the overall survival time, as continued therapy requires a continued survival. Based on our data, we consider it reasonable that the success of the therapy depends on the number of treatment cycles and the corresponding amount of radioactivity cumulatively applied in the long-term run. Corresponding results have been described by Rahbar et al. [4] before, indicating a prolonged survival for patients receiving ≥ 18.8 GBq Lu-177-PSMA-617, which in general is equivalent to three treatment cycles.

In our opinion, patients eligible for systemic Lu-177-PSMA-617-RLT should therefore be selected carefully with respect to the remaining life expectancy, allowing them to receive at least two treatment cycles. Biochemical markers such as LDH, ALP, CRP and GGT may serve as a decision aid. In case of manifest liver metastases Lu-177-PSMA-617-RLT should be considered cautiously and in accordance with the individual burden of disease. Our study is limited by a moderate cohort size and its single-centre design, we belief that further studies are necessary to confirm that these parameters are capable to predict the benefit of Lu-177-PSMA-617-RLT adequately.

## Conclusion

Pre-therapeutic LDH, HB, CRP, ALP and GGT level are helpful parameters for prediction of overall survival of patients receiving Lu-177-PSMA-617-RLT for metastasized prostate cancer. In particular, decreased pre-therapeutic hemoglobin levels and increased LDH levels exceeding the threshold of 250 U/l seem to be associated with poorer survival. Liver metastases indicate a poor prognosis as they have a major impact on survival. Moreover, the overall survival time was strongly associated with the number of treatment cycles applied. A biochemical response might fail to appear during early cycles and should not provoke preliminary withdrawal of the treatment. These findings should be considered by physicians planning systemic radioligand therapies.

## Supplementary Information

**Additional file 1**: **Table S1**. Pre-therapeutic (baseline) serological parameters (n=52). Complete list of obtained blood parameters and related abbreviations.

**Additional file 2**: **Table S2**. Kaplan–Meier estimator depending on previously performed therapies and site of metastatic disease. Calculated Kaplan–Meier estimator depending on previously performed therapies and site of metastatic disease including median survival time and p-values.

**Additional file 3**: **Fig. S1**. Kaplan-Meier plot: Patients w/ preexisting anemia vs. Patients w/o preexisting anemia. Overall survival time subject to the presence of preexisting anemia defined as baseline hemoglobin levels <10g/dl.

**Additional file 4**: **Fig. S2. **Kaplan-Meier plot: Effect of PSA decline on median survival time. Overall survival time compared in patients that presented with a PSA level decrease ≥ 50% after the first therapy cycle versus patients with less than 50% decrease or increasing PSA level (A) and patients with decreasing PSA level of any height vs. patients with increasing PSA level after the first therapy cycle (B).

**Additional file 5**: **Fig. S3**. Kaplan-Meier plot: Patients who received >2 therapy cycles vs. patients who received 1-2 therapy cycles. Difference in survival of patients receiving one or two, or more than two treatment cycles.

## Data Availability

The data that support the findings of this study are not publicly available due to reasons of sensitivity and are available from the corresponding author (UL) upon reasonable request.

## Abbreviations

RLT: Radioligand therapy
mCRPC: Metastasized castration-resistant prostate cancer
PSA: Prostate-specific antigen
OS: Overall survival
LDH: Lactate dehydrogenase
ALP: Alkaline phosphatase
GGT: Gamma-glutamyl transferase
CRP: C-reactive protein
HB: Hemoglobin
HR: Hazard ratio
95%-CI: 95% Confidence interval
*p*: *p*-Value
