# Supplementary material for: Pre- and intratherapeutic predictors of overall survival in patients with advanced metastasized castration-resistant prostate cancer receiving Lu-177-PSMA-617 radioligand therapy
Source: BMC Urol. 2022 Jul 4;22:96. doi: 10.1186/s12894-022-01050-3 (PMC9254582; doi:10.1186/s12894-022-01050-3)
Supplement: Supplementary file 1 — Additional file 1: Table S1. Pre-therapeutic (baseline) serological parameters (n=52). Complete list of obtained blood parameters and related abbreviations. [file 12894_2022_1050_MOESM1_ESM.docx]

**Supp. Table 1** Pre-therapeutic (baseline) serological parameters (n=52).

| **Parameter** | **Unit** | **Median** | **Mean** | **Standard deviation** | **Range** |
| --- | --- | --- | --- | --- | --- |
| Leukocytes | x10^9^/l | 6.04 | 6.36 | 1.99 | 3.29-11.19 |
| Erythrocytes | x10^12^/l | 3.85 | 3.81 | 0.63 | 2.53-5.06 |
| Hemoglobin | g/dl | 11.55 | 11.40 | 1.83 | 7.2-15.4 |
| Hematocrit | % | 33.50 | 33.25 | 5.18 | 23.0-43.1 |
| MCV | fl | 88.30 | 88.05 | 5.43 | 77.9-101.5 |
| MCH | pg | 29.95 | 29.96 | 2.11 | 26.1-34.8 |
| MCHC | g/dl | 34.05 | 34.05 | 1.46 | 29.4-36.9 |
| Platelets | x10^9^/l | 240.50 | 238.31 | 84.07 | 77.0-457.0 |
| MPV | fl | 10.10 | 10.14 | 0.79 | 8.3-12.9 |
| PT | sec | 98.00 | 95.39 | 14.52 | 36.0-120.0 |
| INR | ratio | 1.01 | 1.04 | 0.17 | 0.9-2.03 |
| aPTT | sec | 28.00 | 27.98 | 2.75 | 21.0-38.0 |
| Sodium | mmol/l | 139.50 | 139.56 | 2.70 | 133.0-146.0 |
| Potassium | mmol/l | 4.04 | 4.11 | 0.50 | 3.14-5.42 |
| Chloride | mmol/l | 102.00 | 101.54 | 3.55 | 94.0-110.0 |
| Calcium | mmol/l | 2.34 | 2.32 | 0.12 | 2.03-2.52 |
| Phosphate | mmol/l | 1.00 | 0.99 | 0.22 | 0.34-1.48 |
| Creatinine | μmol/l | 80.50 | 82.00 | 19.81 | 44.0-136.0 |
| GFR | ml/min | 85.00 | 82.76 | 17.60 | 42.0-122.0 |
| Urea | mmol/l | 5.55 | 6.00 | 2.03 | 2.1-11.5 |
| Glucose | mmol/l | 6.07 | 6.16 | 1.17 | 4.69-9.97 |
| Total bilirubin | μmol/l | 5.20 | 5.51 | 2.36 | 2.0-11.3 |
| Uric acid | μmol/l | 274.50 | 298.60 | 110.06 | 152.0-630.0 |
| LDH | U/l | 252.50 | 327.46 | 220.11 | 165.0-1209.0 |
| AST | U/I | 29.35 | 36.26 | 20.78 | 13.9-110.5 |
| ALT | U/I | 16.80 | 21.19 | 15.71 | 6.5-84.3 |
| GGT | U/I | 44.00 | 96.56 | 179.54 | 10.0-1176.0 |
| ALP | U/I | 123.50 | 183.44 | 174.53 | 37.0-951.0 |
| CRP | mg/l | 6.91 | 34.04 | 57.64 | 0.3-262.0 |
| Total protein | g/l | 70.00 | 69.65 | 6.12 | 51.0-81.0 |
| PSA | μg/l | 216.50 | 614.06 | 948.10 | 2.41-4165.0 |

MCV=mean corpuscular volume;MCH=mean corpuscular hemoglobin;MCHC=mean corpuscular hemoglobin concentration;MPV=mean platelet volume;PT=prothrombin time;INR=international normalized ratio;aPTT=activated partial thromboplastin time;GFR= glomerular filtration rate;LDH=lactate dehydrogenase;AST=aspartate aminotransferase;ALT=alanine aminotransferase;GGT=gamma-glutamyl transferase;ALP=alkaline phosphatase;CRP=c-reactive protein;PSA=prostate specific antigen.
