# Supplementary material for: Pre- and intratherapeutic predictors of overall survival in patients with advanced metastasized castration-resistant prostate cancer receiving Lu-177-PSMA-617 radioligand therapy
Source: BMC Urol. 2022 Jul 4;22:96. doi: 10.1186/s12894-022-01050-3 (PMC9254582; doi:10.1186/s12894-022-01050-3)
Supplement: Supplementary file 2 — Additional file 2: Table S2. Kaplan–Meier estimator depending on previously performed therapies and site of metastatic disease. Calculated Kaplan–Meier estimator depending on previously performed therapies and site of metastatic disease including median survival time and p-values. [file 12894_2022_1050_MOESM2_ESM.docx]

**Supp. Table 2** Kaplan–Meier estimator depending on previously performed therapies and site of metastatic disease.

| **Previous therapy** | **Median survival (weeks) when not pre-treated** | **Median survival (weeks) when pre-treated** | **p value** |
| --- | --- | --- | --- |
| Radical prostatectomy | 52.7 (n=19) | 61.0 (n=33) | 0.768 |
| External beam radiation and/or Brachytherapy | 52.7 (n=13) | 55.6 (n=39) | 0.813 |
| Bicalutamide and/or Leuprorelin | 17.9 (n=4) | 55.6 (n=48) | 0.444 |
| Enzalutamide and/or Abiraterone | 116.1 (n=8) | 52.7 (n=44) | 0.018 |
| Chemotherapy (Docetaxel and/or Cabazitaxel) | 103.3 (n=13) | 53.6  (n=39) | 0.091 |
| Ra-223-Dichloride | 55.6 (n=44) | 61.0 (n=8) | 0.689 |
| **Site of metastatic disease** | **Median survival (weeks) when absent** | **Median survival (weeks) when present** | **p value** |
| Lymphatic | 55.6 (n=10) | 61.0 (n=42) | 0.712 |
| Hepatic | 84.4  (n=43) | 28.3 (n=9) | <0.001* |
| Cerebral | 53.6 (n=49) | 64.3  (n=3) | 0.992 |
| Pulmonary | 55.6 (n=49) | 34.0 (n=3) | 0.773 |
