# Supplementary figures and images for: Pre- and intratherapeutic predictors of overall survival in patients with advanced metastasized castration-resistant prostate cancer receiving Lu-177-PSMA-617 radioligand therapy
Source: BMC Urol. 2022 Jul 4;22:96. doi: 10.1186/s12894-022-01050-3 (PMC9254582; doi:10.1186/s12894-022-01050-3)

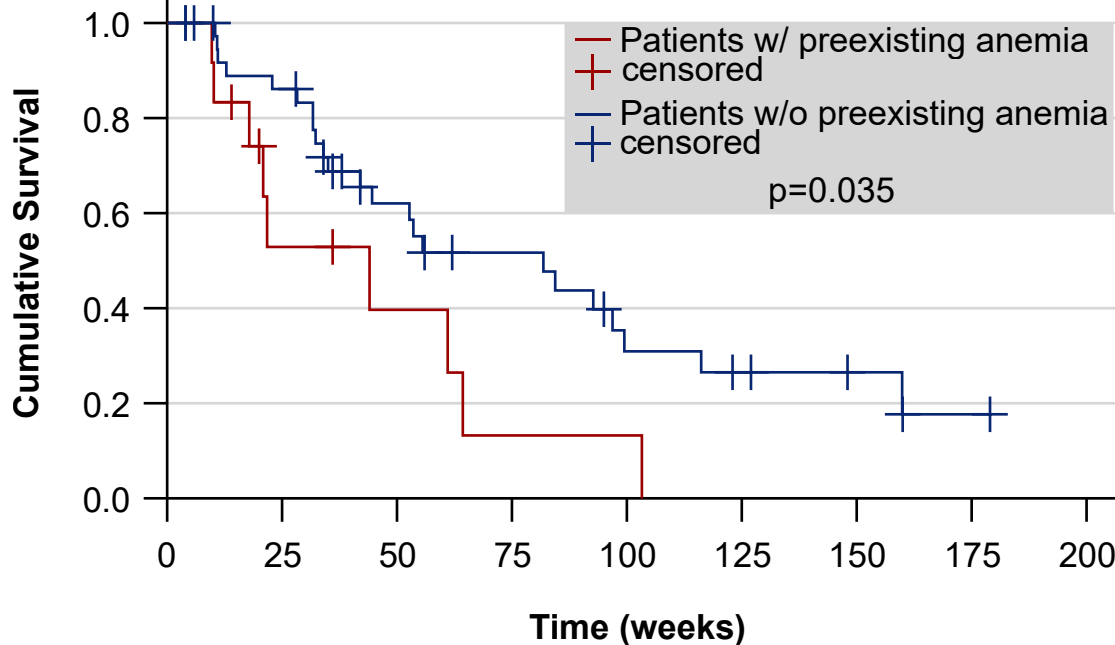

No. at risk:

|   |    |    |    |    |   |   |   |   |   |
|---|----|----|----|----|---|---|---|---|---|
| — | 12 | 5  | 3  | 1  | 1 | 0 | 0 | 0 | 0 |
| — | 40 | 31 | 18 | 13 | 7 | 5 | 3 | 1 | 0 |

Supplement: Supplementary file 3 — Additional file 3: Fig. S1. Kaplan-Meier plot: Patients w/ preexisting anemia vs. Patients w/o preexisting anemia. Overall survival time subject to the presence of preexisting anemia defined as baseline hemoglobin levels <10g/dl. [file 12894_2022_1050_MOESM3_ESM.pdf]

Cumulative Survival

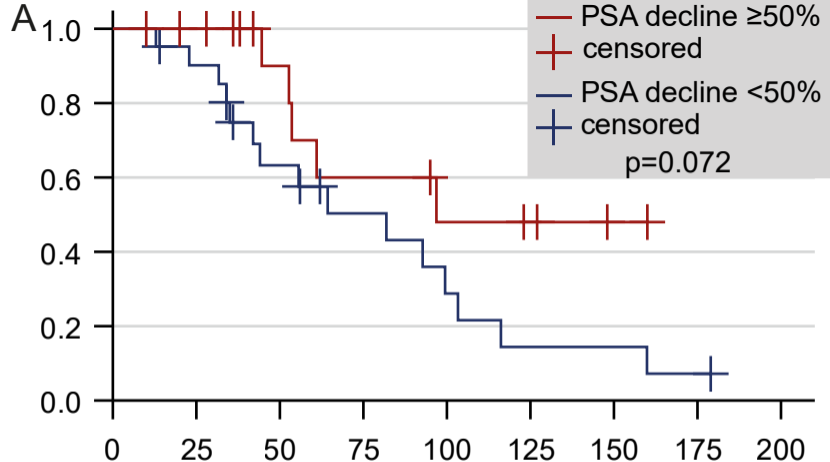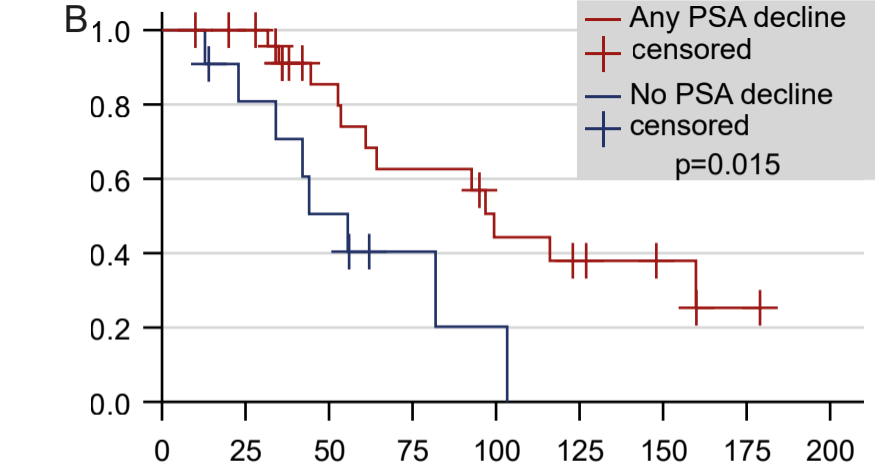

Time (weeks)

Supplement: Supplementary file 4 — Additional file 4: Fig. S2. Kaplan-Meier plot: Effect of PSA decline on median survival time. Overall survival time compared in patients that presented with a PSA level decrease ≥ 50% after the first therapy cycle versus patients with less than 50% decrease or increasing PSA level (A) and patients with decreasing PSA level of any height vs. patients with increasing PSA level after the first therapy cycle (B). [file 12894_2022_1050_MOESM4_ESM.pdf]

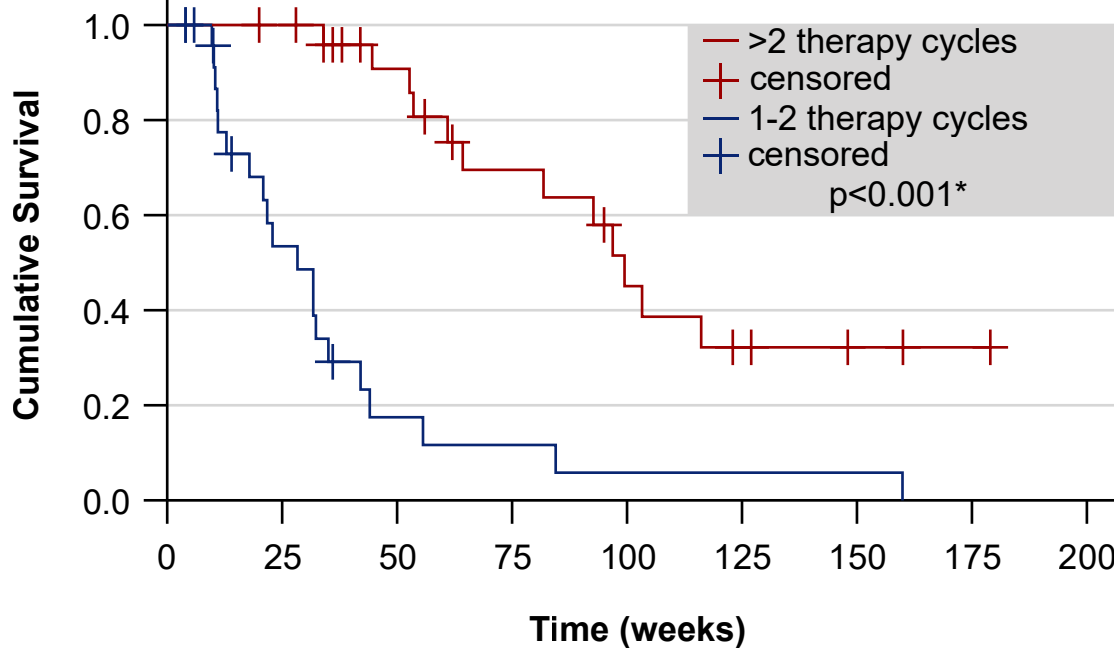

No. at risk:

|   |    |    |    |    |   |   |   |   |   |
|---|----|----|----|----|---|---|---|---|---|
| — | 26 | 25 | 18 | 12 | 7 | 4 | 2 | 1 | 0 |
| — | 26 | 11 | 3  | 2  | 1 | 1 | 1 | 0 | 0 |

Supplement: Supplementary file 5 — Additional file 5: Fig. S3. Kaplan-Meier plot: Patients who received >2 therapy cycles vs. patients who received 1-2 therapy cycles. Difference in survival of patients receiving one or two, or more than two treatment cycles. [file 12894_2022_1050_MOESM5_ESM.pdf]
